# Impact of Helicobacter pylori Infection on Gastric Variceal Bleeding among Patients with Liver Cirrhosis

**DOI:** 10.1155/2019/6529420

**Published:** 2019-02-10

**Authors:** Mohamed A. Elsebaey, Mohamed A. Tawfik, Samah A. Elshweikh, Manal Saad Negm, Mohammed H. Elnaggar, Ghada Mahmoud Alghazaly, Sherief Abd-Elsalam

**Affiliations:** ^1^Internal Medicine Department, Tanta University, Egypt; ^2^Tropical Medicine Department, Tanta University, Egypt

## Abstract

**Background and Aims:**

Currently, it is well known that Helicobacter pylori- (*H. pylori*-) related peptic ulcer is one of the main causes of nonvariceal bleeding in cirrhotic patients. However, there is a lack of data to identify the exact effect of *H. pylori* infection on variceal bleeding. This study was conducted to identify the impact of *H. pylori* infection on gastric variceal bleeding in cirrhotic patients.

**Patients and Methods:**

76 cirrhotic patients with gastric varices were included in this prospective study and divided into 2 groups: nonbleeding gastric varices (32 patients) and bleeding gastric varices (44 patients). The fasting serum gastrin level was measured. Mucosal biopsies from the gastric body and antrum were examined to determine the patterns of gastritis and the presence of *H. pylori*.

**Results:**

The frequency of *H. pylori* infection in the studied patients was 59.2%. There were significant differences between both groups regarding liver decompensation (*P* = 0.001), red color sign over gastric varices (*P* = 0.0011), prevalence of *H. pylori* infection (*P* = 0.0049), histological patterns of gastritis (*P* = 0.0069), and serum gastrin level (*P* = 0.0200). By multivariate analysis, Child C cirrhosis, red color sign over gastric varices, and *H. pylori*-induced follicular gastritis were independent risk factors for bleeding from gastric varices.

**Conclusion:**

*H. pylori*-induced follicular gastritis is considered as an additional risk factor for bleeding from gastric varices.

## 1. Introduction

Gastric variceal bleeding is a serious complication of portal hypertension in cirrhotic patients and is associated with significant morbidity and mortality [1]. Although the incidence and bleeding risk of gastric varices are lower than that of esophageal varices, whenever bleeding occurs, it is usually more severe, requires more blood transfusions, and is associated with higher rebleeding and mortality rates [2–4]. Therefore, the prognosis of patients with gastric variceal bleeding is still far from satisfactory [5].

Most of the cirrhotic patients are immunocompromised; therefore, they are more susceptible to infections, and it seems that there is association between infections and the cirrhosis-related complications such as variceal bleeding [6, 7]. Currently, it is well known that Helicobacter pylori- (*H. pylori*-) related peptic ulcer is one of the main causes of nonvariceal bleeding in cirrhotic patients [8–10]. However, there is a lack of population-based data to identify the exact effect of *H. pylori* infection on gastric variceal bleeding in cirrhotic patients.

There are limited studies that discussed the relation between *H. pylori* infection and variceal bleeding [11]. To address this issue, we conducted this study to assess the effect of *H. pylori* infection on bleeding from gastric varices in cirrhotic patients.

## 2. Patients and Methods

Between January 2017 and May 2018, we performed this prospective study at the gastroenterology and hepatology unit of Internal Medicine Department, Tanta University Hospital, Egypt.

In this study, 298 patients were assessed for enrollment in the study. However, 222 patients were excluded: 10 patients had previous medication for Helicobacter pylori, 31 patients received antibiotics in the last month, 62 patients received proton pump inhibitors in the last 2 weeks, and 119 patients were also excluded due to the presence of isolated esophageal varices. So finally, 76 patients with gastric varices were enrolled in the study.

A total of 76 cirrhotic patients with gastric varices were enrolled in this study. All cirrhotic patients who attended for screening of varices and the endoscope revealed nonbleeding gastric varices and those who presented with upper gastrointestinal bleeding (UGIB) and the endoscope revealed gastric varix as a source of bleeding were recruited in this study. Patients who had previously undergone *H. pylori* treatment or had received proton pump inhibitor (PPI) or antibiotics within the previous 2 or 4 weeks were excluded from the study.

The patients were divided into 2 groups: group I (nonbleeding gastric varices) included 32 patients who attended for variceal screening in which the endoscope revealed nonbleeding gastric varices and group II (bleeding gastric varices) included 44 patients presented with UGIB in whose gastric varix was the source of bleeding.

The study protocol was done in accordance with the ethical guidelines of the 1975 Helsinki Declaration. A written informed consent was obtained from all patients for participation in the current study. Detailed history taking, thorough clinical examination, and routine laboratory investigations were done for all patients. The severity of liver cirrhosis was assessed using Child-Pugh classification [12].

### 2.1. Upper GI Endoscopy and Gastric Biopsy

Endoscopy was done in all patients, and the endoscopic findings of gastric varices such as variceal location, size, and the presence of red color sign were evaluated [13, 14].

Regarding therapy of gastrointestinal bleeding in these patients, patients with variceal bleeding were resuscitated; blood transfusion was given if a hemoglobin level was less than 8 gm/dL. Somatostatin (Sandostatin, Novartis) 100 *μ*g IV as an initial bolus followed by IV infusion of 25 *μ*g/h was administered. Upper endoscopy was done once the patient's vital signs permitted, and haemostatic procedure was achieved using N-butyl-2-cyanoacrylate (ampoule 0.5 mL) (GluStitch® Twist, GluStitch Inc., Delta, BC, Canada) diluted with 0.8 mL of Lipiodol®. Cyanoacrylate was injected using Olympus video endoscopy and a 23-gauge disposable injection needle (Wilson-Cook Medical Inc., USA), immediately followed by injection of 1-2 mL distilled water then the needle was withdrawn [2].

Regarding prophylaxis of gastrointestinal bleeding in these patients, a nonselective *β*-blocker (e.g., propranolol) and repeated endoscopic sessions using cyanoacrylate every 4 weeks are performed until endoscopic obliteration is achieved [5].

In patients with nonbleeding gastric varices (group I), endoscopic biopsies from the gastric corpus and antrum were obtained by biopsy forceps at admission. In patients with bleeding gastric varices (group II), biopsies were obtained during the endoscopic follow-up. The biopsy collected from each patient was kept in 10% formalin to be processed later using haematoxylin/eosin and gimsa stains to determine the patterns of gastritis and the presence of *H. pylori*, respectively.

### 2.2. Fasting Serum Gastrin Level (Normal Level: 13-115 pg/mL)

After an overnight fast, the serum gastrin level was measured by enzyme-linked immunosorbent assay (ELISA) kits provided from Biohit Deutschland GmbH, Germany.

### 2.3. Statistical Analysis

Statistical analysis of data was done using the Statistical Program for Social Science (SPSS) version 20.0. Quantitative data were analyzed using unpaired *t*-test and expressed as mean and standard deviation (SD). Qualitative data were analyzed using the chi-square test and were expressed as frequency and percent. Multivariate analysis was done to identify predictive factors of bleeding gastric varices. In all tests, *P* value was significant if <0.05. (The full detailed form is SPSS 20, IBM, Armonk, NY, United States of America.)

## 3. Results

Regarding demographic data of the studied patients, there were no significant differences between both groups with regard to age, sex, and etiology of cirrhosis (*P* = 0.0940, 0.6387, and 0.6587), respectively, while there was significant difference regarding Child-Pugh class (*P* = 0.001) as shown in Table 1.

Concerning the endoscopic findings of gastric varices, there were no significant differences between both groups regarding type and size of gastric varices (*P* = 0.9427 and 0.6766, respectively), while there was significant difference regarding the red color sign over gastric varices (*P* = 0.0011) as shown in Table 1.

The prevalence of *H. pylori* infection among the studied patients was 59.2%. *H. pylori* infection was significantly more frequent among patients with bleeding gastric varices compared to those without bleeding (*P* = 0.0049). Histopathological patterns of chronic gastritis and the fasting serum gastrin level in both groups were shown in Table 2.

In our study, 12 patients (15.79%) had clean base-peptic ulcers. In the nonbleeding group, 4 patients had peptic ulcers: 2 ulcers at the gastric antrum and other 2 ulcers at the duodenal bulb. However, in the bleeding group, 8 patients had peptic ulcers: 3 ulcers at the gastric antrum and other 5 ulcers at the duodenal bulb.

**Figure 1:**
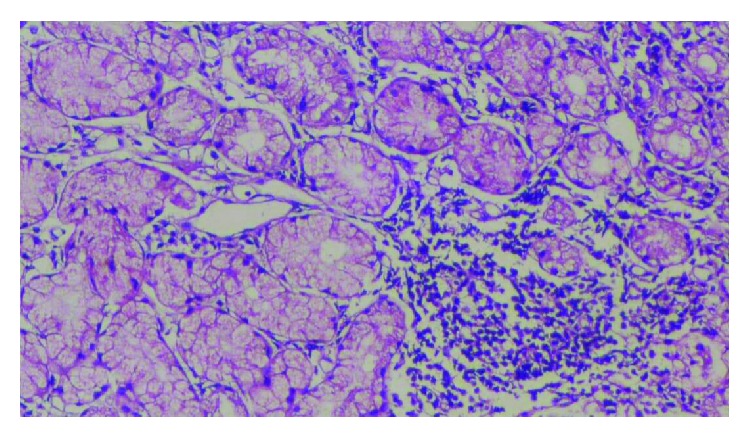
Histopathological pattern of follicular gastritis.

**Figure 2:**
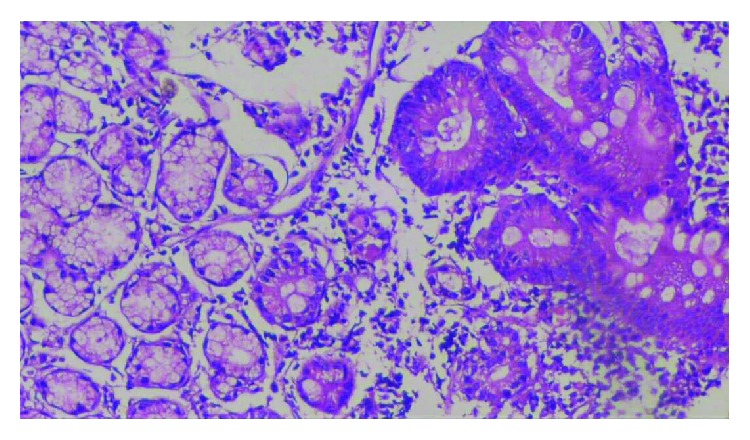
Histopathological pattern of atrophic gastritis.

Histopathological patterns of chronic gastritis and fasting serum gastrin levels among *H. pylori* positive patients were shown in Table 3. In group I (nonbleeding gastric varices), 7 (21.88%) patients had follicular gastritis, while in group II (bleeding gastric varices), 26 (59.09%) patients had follicular gastritis (Figure 1). On the other hand, in group I (nonbleeding gastric varices), 5 (15.63%) patients had atrophic gastritis; in group II (bleeding gastric varices), 2 (4.55%) patients had atrophic gastritis (Figure 2).

By multivariate analysis, Child C cirrhosis, red color sign over gastric varices, and *H. pylori*-induced follicular gastritis were independent risk factors for bleeding from gastric varices in the studied patients as shown in Table 4.

## 4. Discussion

The possible causative role of *H. pylori* infection in gastric variceal hemorrhage is less investigated. Indeed, the known risk factors for gastric variceal bleeding such as hepatic functional reserve, gastric variceal location, variceal size, overlying mucosal red color sign, and intravariceal pressure do not easily explain why variceal bleeding and early rebleeding occur unpredictably in patients with cirrhosis [15–18].

In the current study, the frequency of H. pylori infection was 59.2%. This was consistent with a study of Devrajani et al. [19] who showed that the *H. pylori* infection rate in cirrhotic patients was 56%. In various studies, the overall prevalence of *H. pylori* infection in cirrhotic patients ranged from 35.1% to 70.6%. This discrepancy is perhaps related to the different investigational tools used for the diagnosis of *H. pylori* infection [20–23].

The present study clearly demonstrated that the prevalence of *H. pylori* infection was significantly higher in patients with gastric variceal bleeding than those without bleeding (72.73% and 40.62%, respectively) (*P* = 0.0049). This means that *H. pylori* infection might be implicated as a risk factor for bleeding from gastric varices. In contrary to our results, Sakamoto et al. [11] reported higher rates of *H. pylori* infection in patients without variceal bleeding in comparison to those with bleeding (55.4% and 31.6%, respectively) (*P* = 0.001), indicating that *H. pylori* might have a protective effect against variceal bleeding.

The explanation of this extreme discrepancy between our results and those of Sakamoto et al. [11] might be related to the patterns of gastritis induced by *H. pylori* infection and the concomitant gastric acid secretion. It is well known that chronic *H. pylori* infection can cause atrophic gastritis in some patients and follicular gastritis in the others [24]. In the case of atrophic gastritis, mucosal atrophy is progressively affecting the gastric body and antrum. The gastric acid-producing parietal cells are also atrophied resulting in decreased gastric acid secretion [25]. In the case of follicular gastritis, lymphocytic infiltration involves mainly the gastric antrum and is associated with increased gastrin hormone secretion from G cells that in turn increases gastric acid production from parietal cells [26]. Since the findings of the current study are contradictory to the report from Japan, more studies with larger sample sizes are needed to confirm these findings.

The mechanisms that lead to increased gastrin secretion in *H. pylori*-induced follicular gastritis are unclear. One theory is that local alkalization of ammonia produced by *H. pylori* urease in the vicinity of G cells stimulates gastrin release. Another possible mechanism is that *H. pylori* infection reduces the number of antral D cells and somatostatin concentration, resulting in a lack of physiologic inhibition of somatostatin on G cells and hence increased gastrin secretion [27, 28].

Our results revealed that the rate of follicular gastritis was significantly higher in *H. pylori* positive patients with bleeding gastric varices than those without bleeding (71.88% and 38.46%, respectively) (*P* = 0.0199). This explains why the gastrin level was significantly higher in *H. pylori* positive patients with bleeding gastric varices compared to those without bleeding (78.34 ± 35.2 and 51.15 ± 46.92 pg/mL, respectively) (*P* = 0.0380). On the other hand, Sakamoto et al. [11] documented, in their study, that *H. pylori* infection was commonly associated with atrophic gastritis and concomitant hypoacidity.

On the basis of our findings, we supposed that *H. pylori*-induced follicular gastritis might increase the risk of gastric variceal bleeding through the deleterious effect of gastric hyperacidity associated with hypergastrinemia. From a viewpoint of acid-related concerns, hyperacidity in cirrhotic patients can be a relatively aggressive factor for mucosa-overlying varices causing erosions, ulcerations, and eventually variceal rupture which could be ameliorated by long-term receiving PPI [29].

Several studies reported that the majority of esophageal variceal bleeding that occurred at the distal esophagus near the esophagogastric junction was associated with a decreased esophageal acid clearance. Moreover, the incidence of this bleeding could be reduced by long-term administration of PPI [29–31]. The results of these reports regarding the deleterious effect of hyperacidity on varices, in part, support our results.

To our knowledge, this is the first study to document *H. pylori* infection as a risk factor for bleeding from gastric varices especially if *H. pylori* was associated with follicular gastritis. This could be attributed to hyperacidity associated with follicular gastritis.

In addition, *H. pylori* infection might worsen the liver functions and portal hypertension through overproduction of proinflammatory cytokines such as tumor necrosis factor-*α*, interleukins, nitric oxide, and endothelin-1 [32–38]. This in turn has a harmful effect on the gastric varices especially in patients with advanced liver cirrhosis.

## 5. Conclusion

The prevalence of *H. pylori* infection in cirrhotic patients was 59.2%. In addition to decompensated cirrhosis and red color sign over gastric varices, *H. pylori*-induced follicular gastritis is considered as a risk factor for bleeding gastric varices.

## Figures and Tables

**Table 1:** Demographic data and endoscopic findings of gastric varices in the studied patients.

Variables		Nonbleeding gastric varices (*N* = 32)		Bleeding gastric varices (*N* = 44)		*P* value
		*N*	%	*N*	%	
Age	Mean ± SD	51.03 ± 7.08		53.66 ± 6.35		0.0940
Sex	Male	24	75%	35	79.55%	0.6387
Etiology of cirrhosis	HCV	29	90.63%	38	86.36%	0.6587
	HBV	0	0%	1	2.27%	
	Others	3	9.38%	5	11.36%	
Child-Pugh class	A	7	21.88%	4	9.09%	**0.001** ^∗^
	B	17	53.13%	10	22.73%	
	C	8	25%	30	68.18%	
Types of gastric varices	GOV1	20	62.5%	26	59.09%	0.9427
	GOV2	9	28.13%	13	29.55%	
	IGV1	3	9.38%	5	11.36%	
Size of gastric varices	Small	4	12.5%	7	15.91%	0.6766
	Large	28	87.5%	37	84.09%	
Red color sign over gastric varices	Yes	9	28.13%	29	65.91%	**0.0011** ^∗^
	No	23	71.88%	15	34.09%	

^∗^GOV1: gastroesophageal varices type 1; GOV2: gastroesophageal varices type 2; IGV1: isolated gastric varices type 1.

**Table 2:** Prevalence of *H. pylori* infection, histopathological patterns of chronic gastritis, and the fasting serum gastrin level among the studied patients.

Variables		Nonbleeding gastric varices (*N* = 32)		Bleeding gastric varices (*N* = 44)		*P* value
		*N*	%	*N*	%	
*H. pylori* infection	Positive	13	40.62%	32	72.73%	**0.0049** ^∗^
	Negative	19	59.38%	12	27.27%	
Histopathological patterns	Follicular	7	21.88%	26	59.09%	**0.0069** ^∗^
	Atrophic	5	15.63%	2	4.55%	
	Erosive	9	28.13%	10	22.73%	
	Superficial	11	34.38%	6	13.64%	
Fasting serum gastrin level	Mean ± SD	48.19 ± 30.675		66.93 ± 36.085		**0.0200** ^∗^

**Table 3:** Histopathological patterns of chronic gastritis and the fasting serum gastrin level among H. pylori positive patients.

Variables		Nonbleeding gastric varices (*N* = 13)		Bleeding gastric varices (*N* = 32)		*P* value
		*N*	%	*N*	%	
H. pylori-induced chronic gastritis	Follicular	5	38.46%	23	71.88%	**0.0199** ^∗^
	Atrophic	5	38.46%	2	6.25%	
	Others (superficial or erosive)	3	23.08%	7	21.88%	
Fasting serum gastrin level	Mean ± SD	51.15 ± 46.92		78.34 ± 35.2		**0.0380** ^∗^

**Table 4:** Multivariate analysis of predictors of bleeding gastric varices among the studied patients.

Variables	Multivariate		
	Adjusted OR	95% CI	*P* value
Decompensated liver cirrhosis (Child-Pugh C)	5.325	2.947–9.159	**0.001** ^∗^
Red color sign over gastric varices	4.941	1.3834–13.312	**0.001** ^∗^
H. pylori infection	3.897	1.480–10.265	**0.005** ^∗^
Histopathological patterns of chronic gastritis	6.527	2.843–13.582	**0.037** ^∗^
Fasting serum gastrin level	5.321	0.259–12.521	0.187

## Data Availability

The authors' institution does not allow public data access.

## References

[1] Haq I., Tripathi D. (2017). Recent advances in the management of variceal bleeding. *Gastroenterology Report*.

[2] Garcia-Tsao G., Sanyal A. J., Grace N. D., Carey W., Practice Guidelines Committee of the American Association for the Study of Liver Diseases, Practice Parameters Committee of the American College of Gastroenterology (2007). Prevention and management of gastroesophageal varices and variceal hemorrhage in cirrhosis. *Hepatology*.

[3] D’Amico G., de Frabchis R. (2003). Upper digestive bleeding in cirrhosis. Post-therapeutic outcome and prognostic indicators. *Hepatology*.

[4] Sarin S. K., Negi S. (2006). Management of gastric variceal haemorrhage. *Indian Journal of Gastroenterology*.

[5] Garcia-Pagán J. C., Barrufet M., Cardenas A., Escorsell À. (2014). Management of gastric varices. *Clinical Gastroenterology and Hepatology*.

[6] Gan Z. H., Tsai C. C., Tseng K. C., Tsai C. C., Hsieh Y. H., Hung T. H. (2014). The effect of bacterial infections in cirrhotic patients with esophageal variceal bleeding. *Annals of Hepatology*.

[7] Thalheimer U., Triantos C. K., Samonakis D. N., Patch D., Burroughs A. K. (2005). Infection, coagulation, and variceal bleeding in cirrhosis. *Gut*.

[8] Eid K.-A., Shawky M. E.-G., Hassan A. M., Mohammed A. Q., Mohammed M. I. (2016). Prevalence of *Helicobacter pylori* infection in patients with portal hypertensive gastropathy owing to liver cirrhosis in Upper Egypt. *Al Azhar Assiut Medical Journal*.

[9] Chen C. T., Wang T. F., Chan C. C. (2002). Role of chronic Helicobacter pylori infection in hyperdynamic circulation of cirrhotic patients. *Hepato-Gastroenterology*.

[10] Kirchner G. I., Beil W., Bleck J. S., Manns M. P., Wagner S. (2011). Prevalence of *Helicobacter pylori* and occurrence of gastroduodenal lesions in patients with liver cirrhosis. *International Journal of Clinical and Experimental Medicine*.

[11] Sakamoto Y., Oho K., Toyonaga A. (2013). Effect of *Helicobacter pylori* infection on esophagogastric variceal bleeding in patients with liver cirrhosis and portal hypertension. *Journal of Gastroenterology and Hepatology*.

[12] Pugh R. N. H., Murray-Lyon I. M., Dawson J. L., Pietroni M. C., Williams R. (1973). Transection of the oesophagus for bleeding oesophageal varices. *The British Journal of Surgery*.

[13] Sarin S. K., Lahoti D., Saxena S. P., Murthy N. S., Makwana U. K. (1992). Prevalence, classification and natural history of gastric varices: a long-term follow-up study in 568 portal hypertension patients. *Hepatology*.

[14] Tajiri T., Yoshida H., Obara K. (2010). General rules for recording endoscopic findings of esophagogastric varices (2nd edition by The Japan Society for Portal Hypertension). *Digestive Endoscopy*.

[15] Park E. J., Jang J. Y., Lee J. E. (2013). The risk factors for bleeding of fundal varices in patients with liver cirrhosis. *Gut and Liver*.

[16] Nafeh H. M., Swifee Y. M., El-Khayat H. R., Bzeed S. E. (2013). Gastric varices: frequency and risk factors for bleeding in Upper Egypt portal hypertension patients. *Al-Azhar Assiut Medical Journal*.

[17] Kim T., Shijo H., Kokawa H. (1997). Risk factors for hemorrhage from gastric fundal varices. *Hepatology*.

[18] Mahl T. C., Groszmann R. J. (1990). Pathophysiology of portal hypertension and variceal bleeding. *The Surgical Clinics of North America*.

[19] Devrajani B. R., Devrajani T., Kumar R., Shah S. Z., Memon A. S. (2010). *Helicobacter pylori* infection in cirrhotic patients with upper gastrointestinal bleeding. *World Applied Sciences Journal*.

[20] Pogorzelska J., Łapińska M., Kalinowska A., Łapiński T. W., Flisiak R. (2017). Helicobacter pylori infection among patients with liver cirrhosis. *European Journal of Gastroenterology & Hepatology*.

[21] Kim D. J., Kim H. Y., Kim S. J. (2008). *Helicobacter pylori* infection and peptic ulcer disease in patients with liver cirrhosis. *The Korean Journal of Internal Medicine*.

[22] Queirvoz D. M. M., Rocha A. M. C., Rocha G. A. (2006). Association between *Helicobacter pylori* infection and cirrhosis in patients with chronic hepatitis C virus. *Digestive Diseases and Sciences*.

[23] Lo G. H., Yu H. C., Chan Y. C. (2005). The effects of eradication of *Helicobacter pylori* on the recurrence of duodenal ulcers in patients with cirrhosis. *Gastrointestinal Endoscopy*.

[24] Dixon M. F., Genta R. M., Yardley J. H., Correa P. (1996). Classification and grading of gastritis. The updated Sydney System, international workshop on the histopathology of gastritis, Houston 1994. *The American Journal of Surgical Pathology*.

[25] Iijima K., Sekine H., Koike T., Imatani A., Ohara S., Shimosegawa T. (2005). Serum pepsinogen concentrations as a measure of gastric acid secretion in *Helicobacter pylori*-negative and -positive Japanese subjects. *Journal of Gastroenterology*.

[26] McColl K. E. L., El-Omar E., Gillen D. (1998). Interactions between *H. pylori* infection, gastric acid secretion and anti-secretory therapy. *British Medical Bulletin*.

[27] Waldum H. L., Kleveland P. M., Sørdal Ø. F. (2016). *Helicobacter pylori* and gastric acid: an intimate and reciprocal relationship. *Therapeutic Advances in Gastroenterology*.

[28] Park S. M., Yoo B. C., Lee H. R., Yoon J. H., Cha Y. J. (1993). Antral Helicobacter pylori infection, hypergastrinemia and peptic ulcers: effect of eradicating the organism. *The Korean Journal of Internal Medicine*.

[29] Nishiki R., Kuwayama H., Suzuki K. (2005). Randomized controlled trial of long-term proton pump inhibitor (PPI) in the prevention of esophageal variceal bleeding in cirrhotic patients. *Gastroenterology*.

[30] Hidaka H., Nakazawa T., Wang G. (2012). Long-term administration of PPI reduces treatment failures after esophageal variceal band ligation: a randomized, controlled trial. *Journal of Gastroenterology*.

[31] Iwakiri K., Kobayashi M., Sesoko M., Nomura T. (1993). Gastroesophageal reflux and esophageal motility in patients with esophageal varies. *Gastroenterologia Japonica*.

[32] Licinio R., Losurdo G., Carparelli S. (2016). *Helicobacter pylori*, liver cirrhosis, and portal hypertension: an updated appraisal. *Immunopharmacology and Immunotoxicology*.

[33] Waluga M., Kukla M., Żorniak M., Bacik A., Kotulski R. (2015). From the stomach to other organs: *Helicobacter pylori* and the liver. *World Journal of Hepatology*.

[34] Patel M. K., Trombly M. I., Kurt-Jones E. A. (2012). Innate immune responses to *Helicobacter pylori* infection: an overview. *Methods in Molecular Biology*.

[35] Abbas Z., Yakoob J., Usman M. W., Shakir T., Hamid S., Jafri W. (2014). Effect of *Helicobacter pylori* and its virulence factors on portal hypertensive gastropathy and interleukin (IL)-8, IL-10, and tumor necrosis factor-alpha levels. *Saudi Journal of Gastroenterology*.

[36] El-Kalla F., Mansour L., Kobtan A. (2018). Blood ammonia level correlates with severity of cirrhotic portal hypertensive gastropathy. *Gastroenterology Research and Practice*.

[37] Shehata M. A. H., Talaat R., Soliman S., Elmesseri H., Soliman S., Abd-Elsalam S. (2017). Randomized controlled study of a novel triple nitazoxanide (NTZ)-containing therapeutic regimen versus the traditional regimen for eradication of *Helicobacter pylori* infection. *Helicobacter*.

[38] Abd-Elsalam S., Kobtan A., El-Kalla F. (2016). A 2-week nitazoxanide-based quadruple treatment as a rescue therapy for *Helicobacter pylori* eradication: a single center experience. *Medicine*.

